# A novel dance intervention program for children and adolescents with developmental disabilities: a pilot randomized control trial

**DOI:** 10.1186/s13102-024-00897-3

**Published:** 2024-05-14

**Authors:** Jeffrey T. Anderson, Christina Toolan, Emily Coker, Hannah Singer, Derek Pham, Nicholas Jackson, Catherine Lord, Rujuta B. Wilson

**Affiliations:** 1 University of California, Los Angeles, Los Angeles, CA USA; 2 California State University, Dominguez Hills, Carson, CA USA; 3 Stanford School of Medicine, Stanford, CA USA; 4 Semel Institute for Neuroscience and Human Behavior, 760 Westwood Plaza, Los Angeles, CA 90024 USA

**Keywords:** Disability, Motor, Dance, Intervention, Developmental, Randomized Control Trial

## Abstract

**Background:**

Organized physical activity programs have been shown to provide wide benefits to participants, though there are relatively few studies examining the impact of these programs for individuals with developmental disabilities. This pilot study was conducted to determine the feasibility and impact of an undergraduate-led dance intervention program for children and adolescents with developmental disabilities. We evaluated the impact of the dance program on motor ability and social skills.

**Methods:**

The study design was a waitlist control clinical trial in which participants were randomized to active and control groups. Eligible participants included male and female children and adolescents between the ages of 4 and 17 years with neurodevelopmental disabilities. The Motor Assessment Battery for Children Checklist and the Social Responsiveness Scale were used to assess change in motor and social skills, respectively. After gathering baseline data, the active group completed 1 h of online dance classes per week for 10 weeks, while the control group entered a 10-week waiting period. All participants then returned for a follow-up visit. Pre- and post-intervention data were analyzed using linear mixed-effects modeling adjusting for age and class attendance with subject random intercept.

**Results:**

We recruited and randomized 43 participants with neurodevelopmental disabilities (mean age = 8.63, SD = 2.98), of which 30 participated in dance classes. The attendance rate was 82.6% for the active group and 61.7% for the control group. The active group demonstrated a significant improvement in motor skills in an unpredictable environment, as indicated on the Motor Assessment Battery for Children Checklist (*n* = 21, *p* = 0.05). We also observed positive trends in social skills that did not reach significance.

**Conclusions:**

Our results indicate that it is feasible to develop and implement a fully digital dance intervention program for individuals with developmental disabilities. Further, we find that change in motor skills can be detected after just 10 h of low-intensity participation. However, a lack of significant change in social skills coupled with limitations in study implementation suggests further research is needed to determine the full impact of this dance program.

**Trial Registration:**

ClinicalTrials.gov Protocol Registration System: Protocol ID 20-001680-AM-00005, registered 17/2/2021 – Retrospectively Registered, https://clinicaltrials.gov/study/NCT04762290.

## Background

Organized physical activity (OPA), which is structured physical activity led by a coach or instructor, has wide benefits for physical health and wellbeing. It is well established that routine physical activity reduces risk for multiple chronic conditions and improves health outcomes [1]. Physical activity is also associated with the formation of fundamental motor skills in early childhood, highlighting its importance for motor development [2]. The World Health Organization echoed the importance of daily physical activity for children and adolescents to strengthen muscles and reduce sedentary behavior [3]. In addition, physical activity may provide benefits to the psychological wellbeing of adolescents through strengthening cognitive function networks in the brain [4]. Importantly, not all types of physical activity provide the same array of benefits; this distinction, however, has not yet been thoroughly explored. One study which investigated the relationship between structured and unstructured physical activity found that structured physical activity with guided opportunities for practice proved to be the most beneficial for motor skill development [5]. Similarly, structured indoor and outdoor activities have been shown to reduce the yearly increase of body mass index for developing children. Researchers have found a smaller increase in BMI during what is described as the adiposity rebound period of childhood for children who participate in these activities compared to those who do not [6]. These findings illustrate that within the broader context of physical activity, participation in OPA is a particularly effective way for children and adolescents to improve their physical health and wellbeing.

Despite OPA’s known benefits, much of the research around OPA focuses on typically developing children. Comparatively fewer studies investigated the benefits of OPA programs for children with neurodevelopmental disabilities (NDD), despite the fact that these children often face greater barriers to participation in physical activity. NDD, as defined in the Diagnostic and Statistical Manual, 5th edition, refers to a group of conditions including autism, attention deficit/hyperactivity disorder (ADHD), and cerebral palsy, that often emerge before grade school and are characterized by developmental deficits in personal, social, academic, or occupational domains [7]. Furthermore, it is not uncommon for children and adolescents to be diagnosed with more than one NDD [8]. Literature has shown that adolescents with NDDs are less likely to engage in OPA than neurotypical peers [9–11]. It is well known that individuals with NDDs often have difficulties in physical movement and mobility. In autistic patients, this can include as praxis, object manipulation, and postural stability [12], while cerebral palsy is characterized by high muscle tone and missed motor milestones. Motor challenges also have broad negative impacts on adaptive function and quality of life. Adolescents with cerebral palsy have reported higher physical quality of life, social quality of life, and overall happiness when able to be more physically active [13]. Likewise, motor difficulties in autism are negatively correlated with social skills [14]. This is significant because social challenges can lead to further barriers, including negative social interactions with peers [15] and higher feelings of loneliness [16].

In addition, there is a lack of programs led by physical education coaches with the training and knowledge to adapt the program to individual needs. Limited education for coaches regarding disability has a significant impact on the number of available and adequately trained coaches, and may negatively affect disabled individuals’ participation in sports and other forms of physical activity [17]. Semi-structured interviews with coaches of autistic athletes have shown that the coach-athlete relationship is a particularly important theme, suggesting that adapting teaching styles according to the experience of autistic individuals is an effective coaching strategy [18]. While research into this topic is sparse, these findings offer evidence that encouraging coaches to adopt adaptive teaching styles may reduce barriers to participating in physical activity programs for individuals with NDDs.

There are several examples of physical activity programs which have been successfully implemented for individuals with NDDs and have shown benefits across domains. Previous research has shown that following participation in group OPA programs, autistic children had improved overall motor skills, including aiming, catching, and balance, as well as improved social communication and social motivation [19, 20]. Another review of movement interventions for children with intellectual disabilities found improvements in fundamental motor skills and balance [21]. Researchers have also explored dance-based OPA programs as interventions for children with NDDs, and found techniques such as mirroring and exploratory movement to benefit social and communication skills, motor skills, and behavioral domains [22, 23]. The success of these programs demonstrates the need to further reduce the barriers individuals with NDDs face by developing and evaluating new OPA programs that are adapted for their needs.

To address this gap, we established an organized dance intervention program called the Expressive Movement Initiative (EMI) at the University of California, Los Angeles (UCLA). The course model was designed to achieve meaningful participation for each dancer by creating an adaptable framework which acknowledges individual needs. To achieve that goal, each dancer was paired individually with an undergraduate buddy who had been trained about NDDs, inclusive language, neurodiversity, and adaptive dance and movement teaching styles. Using a strength-based approach [18, 24], this framework ensured that each dancer had the support of an individual who was equipped to support their needs. Furthermore, the use of student buddies to carry out the program greatly increased the feasibility of maintaining one-to-one pairings which in turn provided adequate support for participants and increased opportunities for meaningful social interactions. A document detailing the structure of the dance classes and the protocol for the study at the time of this publication can be found on the clinicaltrials.gov website (https://clinicaltrials.gov/study/NCT04762290?tab=history&a=3). Here we present interim analysis of our study protocol. We choose to present interim analysis due to impacts of the COVID-19 global pandemic on the delivery of the intervention and participant retention. Our goals are to present [1] the feasibility of developing and implementing the EMI program, and [2] the results of two of our standardized outcome measures that were not affected by data attrition. This includes one primary outcome measure – the Motor Assessment Battery for Children Checklist (mABC-C), and one secondary outcome measure – the Social Responsiveness Scale (SRS). Other measures described in our study protocol are not presented due to attrition that resulted in incomplete datasets and will be presented when the full sample is collected and complete. We hypothesized that participants would show improvements in motor and social skills following participation in this program as indicated by the mABC-C and SRS, respectively.

## Methods

Our study design and research methods were reviewed and approved by the University of California, Los Angeles Institutional Review Board (IRB#20-001680). Due to the age of the participant population and/or diagnoses that affect cognitive abilities, a legally authorized representative of all participants provided written informed consent for their data to be used in related research.

### Participants

Eligible participants were between the ages of 4 and 17 with a diagnosed NDD from a healthcare provider, which was reported by parents during eligibility screening. Exclusion criteria was previous participation in EMI dance classes. There were no exclusion criteria related to the degree of intellectual/physical disability or co-occurring health conditions. Participants living in the United States were recruited for this study through flyers and social media listings. Interested families contacted the study team directly and all prospective participants were screened until recruitment goals were met. In instances where parents reported more than one diagnosed NDD, e.g., autism and co-occurring ADHD, or a NDD with a co-occurring condition that falls into a different diagnostic category, e.g., anxiety, this information was recorded onto the participant ID key by a researcher at study entry. Furthermore, parents were asked to report racial and ethnic affiliation.

All potential participants were screened in January and February of 2021. Pre-testing took place from February 15, 2021 to March 5, 2021 and follow-up visits occurred from May 2, 2021 to May 9, 2021. The trial, which was originally planned to conclude in March 2024, was interrupted after one year to perform an interim analysis on the data collected during the COVID-19 pandemic. This decision was made in order to perform a feasibility assessment and adjust the study protocol to include direct in-person measures upon resuming the trial.

### Intervention design

**Fig. 1:**
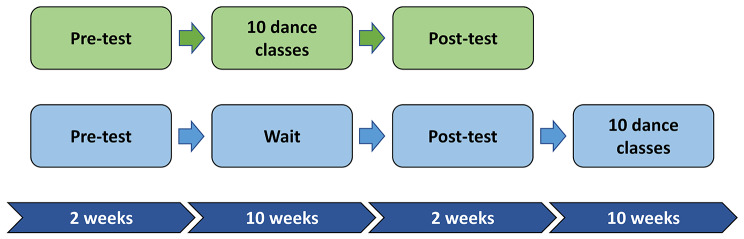
Graphical representation of the longitudinal study design. *The timeline for study events as they relate to the dance intervention*

The intervention was designed as a longitudinal waitlist-control study in which participants were randomized into active and control groups using permuted block randomization with a 2:1 active to control randomization scheme. Study data were collected and managed using Research Electronic Data Capture (REDCap) hosted at UCLA [25]. REDCap is a secure, web-based software platform designed to support data capture for research studies, providing (1) an intuitive interface for validated data capture; (2) audit trails for tracking data manipulation and export procedures; (3) automated export procedures for seamless data downloads to common statistical packages; and (4) procedures for data integration and interoperability with external sources. REDCap was also used to complete the randomization, with the allocation sequence being generated by author six and participants being enrolled by author eight. The randomization list was concealed by REDCap, with the assignment group only being known once an intervention group was assigned. Additionally, participants were stratified by language level (complex speech, phrased speech, or minimally verbal) in order to ensure an even distribution of baseline communication skills. The treatment period was 10 weeks with weekly 1-hour classes. The active and control group completed pre and post intervention surveys online via Zoom in an interview format. Whereas participants were aware of which group they had been placed in, assessments were conducted by trained research staff who were blinded to assignment group. The control group was offered participation in the dance classes after the post-intervention data collection (Fig. 1).

The EMI dance intervention classes were carried out by trained undergraduate students. Each session consisted of an artistic director to lead the class and buddies that were paired 1:1 to each participant. Buddies received training around NDD, accessible language, and how to adapt their teaching for their buddy. This training included attending speaker presentations and reviewing weekly feedback provided by a class instructor. Due to the COVID-19 pandemic, all classes were held virtually on Zoom. To ensure the quality of virtual instruction, artistic directors and buddies received training in adapting movements for Zoom delivery. Additionally, artistic directors provided buddies with written feedback on a weekly basis to provide strategies for adjusting teaching styles to their dancer.

**Fig. 2:**
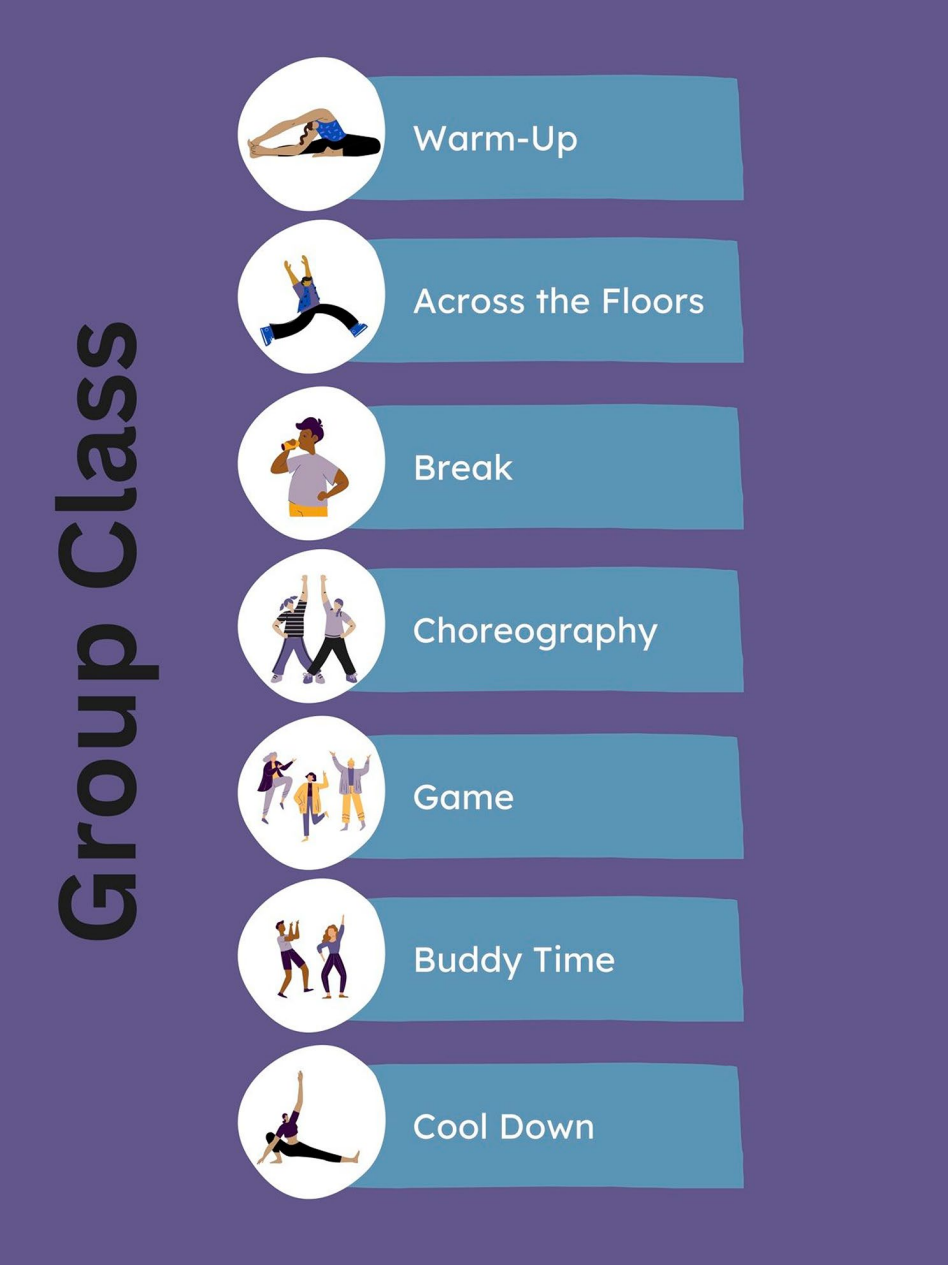
Group Class Agenda. *The typical order of activities that occur during group class. During buddy class, dancers would spend additional time with their buddy in lieu of learning new choreography or playing a game*

The structure of each class could take one of two forms, group class or buddy class, which alternated each week. Dancers started each group class with a warm up consisting of stretches and other movements, followed by an “across the floors” exercise which involved more exaggerated movement. Across the floor movements were commonly based in ballet techniques, such as “relevé walks”, “step, prep, passé,” and “reach chassé.” A short break was incorporated into every class to encourage hydration and resting. After the break, the instructor taught a short choreography to the dancers, which would first be practiced at a pace best for the participant before being paired with music. To close out group time, a musical game was played. These games would require dancers to follow a particular objective, such as balance a tissue on their head while dancing, or follow instructions embedded within a song, such as the hokey-pokey. Following this, dancers were sent into Zoom breakout rooms for a few minutes to work with their buddies. Upon their return, they were given the opportunity to share what they did with their buddy and engage in a cooldown before ending class. A visual representation of a typical group class can be seen in Fig. 2.

Buddy classes began with a warm up and an across the floors series similar to group class. However, after this point they would be sent directly into breakout rooms with their buddies for the remainder of class, which usually lasted 40–45 min. Class plans would provide buddies with objectives to accomplish during their one-on-one session, such as play a musical game or come up with specific dance skills to practice. This structure allowed dancers to receive more individualized attention and work on learned movement skills. Much like group classes, buddy classes ended with dancers coming back to the main room, sharing what they did with their buddy, and engaging in a cooldown activity.

### Measures used pre- and post-intervention

*Social Skills*: Social skills were assessed through parent responses to the Social Responsiveness Scale 2nd edition (SRS-2) [26]. The SRS-2 is a continuous measure of social behaviors that is normed and validated for use across the lifespan in autistic individuals as well as non-autistic individuals who may show various impairments. It captures behaviors related to 5 subscales: (a) social awareness, (b) social cognition, (c) social communication, (d) social motivation, and (e) restricted interested and repetitive behavior. The instrument contains 65 items that took parents approximately 15 min to complete.

*Motor Function.* Motor function was assessed via parent responses to the Movement Assessment Battery for Children Checklist (mABC-C) [27]. This checklist is intended to be completed by a teacher or parent and contains questions pertaining to a variety of motor tasks. The mABC-C contains 30 items, which took parents approximately 10 min to complete. It is validated for use in children ages 5 through 12 with or without motor challenges as a screening tool for Developmental Coordination Disorder (DCD) [28, 29]. The instrument yields a total motor score, wherein a higher score indicates worse motor skills as more characteristics meet criteria for DCD. The mABC-C is associated with a direct assessment battery, the Movement Assessment Battery for Children –second edition (mABC-2), which is commonly used as a tool for assessing children with suspected motor skill impairment [30]. As we were unable to conduct in-person visits due to the COVID-19 pandemic, the mABC-C was used to target relevant motor domains.

### Statistical analysis

The primary analysis was intention-to-treat and included all randomly assigned participants who completed the mABC-C and the SRS in at least one study visit. Dance class attendance rate was calculated independently for the active and control groups. Linear mixed-effects modeling adjusted for age and class attendance with subject random intercept was used to evaluate the change in motor function and social skill scores after undergoing EMI dance classes. Between group differences in change over time were assessed using a group-by-time interaction term. All statistical significance was determined using a two-sided alpha level less than 0.05, and all analyses were conducted using R Version 4.2.1 [31].

## Results

A total of 43 participants were recruited and randomized for this study, of which 61 percent were male. Speech level in this sample ranged from non-speaking to fully-verbal. All participants had a diagnosed NDD, with a majority of participants having a diagnosis of autism (Table 1). A summary of parental racial and ethnic affiliation can be seen in Table 2.

**Table 1:** Demographic and Diagnostic Characteristics of Participants at Baseline

Baseline characteristic	Full sample		Active		Wait-list control		*p*-value
	*n*	%	*n*	%	*n*	%	
Gender							0.99
Female	16	39.0	9	37.5	7	41.2	
Male	25	61.0	15	62.5	10	58.8	
Diagnosis							
Autism	33	80.5	20	83.3	13	76.5	0.70
ADHD/ADD	13	31.7	9	37.5	4	23.5	0.50
Anxiety	6	14.6	3	12.5	3	17.6	0.68
Sensory Processing Disorder	6	14.6	3	12.5	3	17.6	0.68
Speech delay/disorder	6	14.6	5	20.8	1	5.9	0.37
Cerebral palsy	5	12.2	3	12.5	2	11.8	0.99
Developmental delay	2	4.9	1	4.2	1	5.9	0.99
Genetics syndrome	4	9.8	1	4.2	3	17.6	0.29
Other ^a^	8	19.5	3	12.5	5	29.4	0.24
Speech Level							0.50
Complex speech	24	58.5	16	66.7	8	47.1	
Phrased speech	11	26.8	5	20.8	6	35.3	
Minimally verbal	6	14.6	3	12.5	3	17.6	
Age (years)							0.06
4–7	20	48.8	8	33.3	12	70.6	
Male	11	26.8	5	20.8	6	35.3	
Female	9	22.0	3	12.5	6	35.3	
8–12	16	39.0	12	50.0	4	23.5	
Male	10	24.4	7	29.2	3	17.6	
Female	6	14.6	5	20.8	1	5.9	
13–17	5	12.2	4	16.7	1	5.9	
Male	4	9.8	3	12.5	1	5.9	
Female	1	2.4	1	4.2	0	0.0	

Table 1 N *= 41. Two participants were reported to have characteristics of autism but did not have a formal diagnosis. Two participants who were recruited for the study are not included in this table, as they dropped out prior to consenting.*^*a*^*Includes apraxia, hypotonia, epilepsy, oppositional defiant disorder, and depression. All* *p* *values were calculated using Fisher’s Exact Test*

**Table 2:** Parental Racial and Ethnic Affiliation

	Mother		Father	
	*n*	%	*n*	%
Ethnic Affiliation				
Hispanic or Latin Decent	3	6.98	4	9.30
Not of Hispanic or Latin Descent	16	37.21	15	34.88
Decline to Answer	2	4.65	2	4.65
Unreported	22	51.16	22	51.16
Racial Affiliation				
American Indian/Alaska Native	1	2.33	1	2.33
Asian	4	9.30	6	13.95
Native Hawaiian or Pacific Islander	0	0.00	0	0.00
Black or African American	0	0.00	0	0.00
White	15	34.88	13	30.23
Other	0	0.00	0	0.00
Decline to Answer	1	2.33	1	2.33
Unreported	22	51.16	22	51.16

Table 2 N *= 43. Categories are in line with reporting requirements of the funding agency that supported this study*

**Fig. 3:**
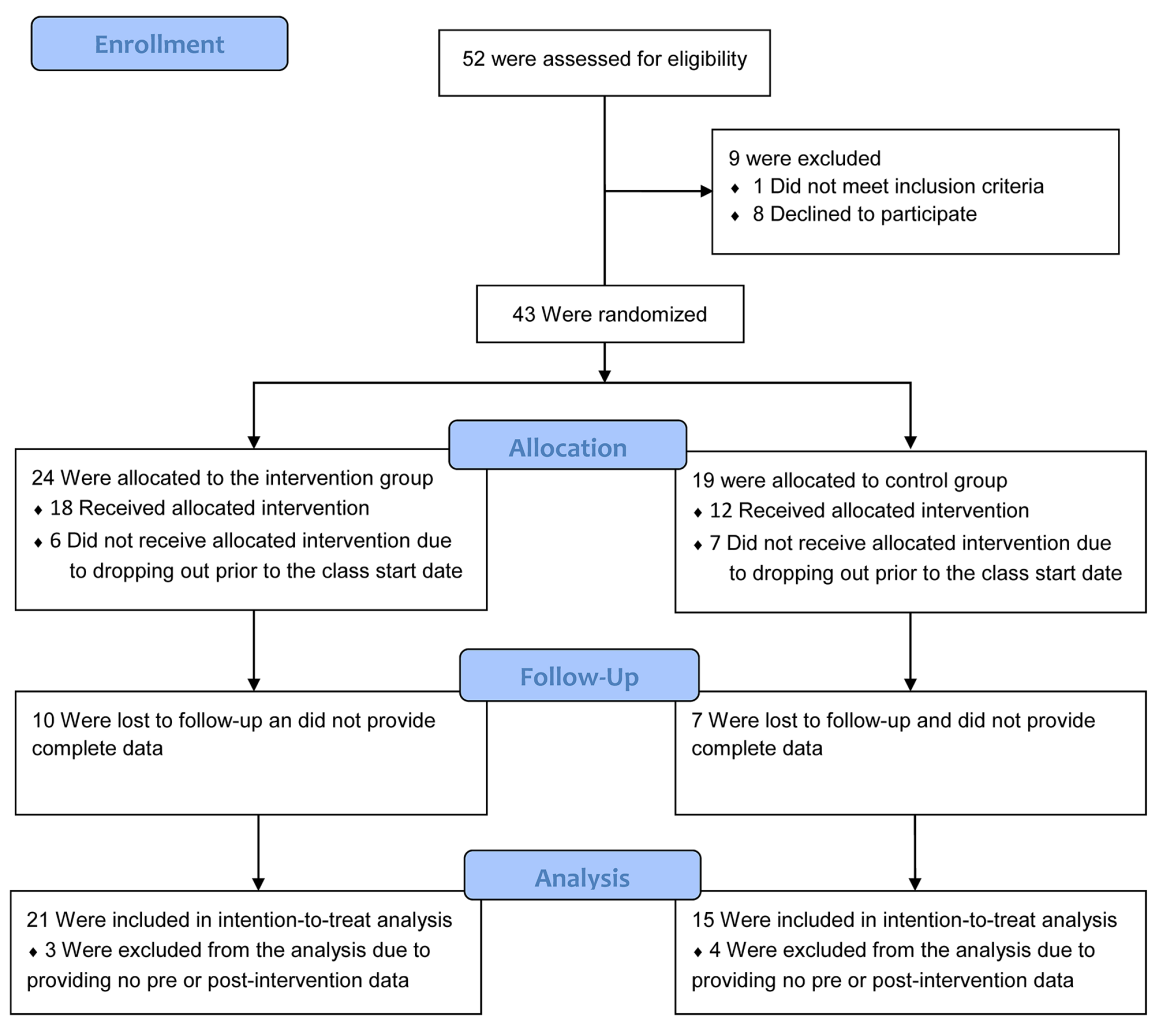
Participant Flow Diagram. Figure 3*Shows the number of participants that were screened, randomized, and ultimately included in the analysis. A power analysis was performed on the full sample size projected over the multi-year study. No power analysis was performed on the sample reported in this interim analysis*

Out of the 43 participants who were allocated to an intervention group, 36 completed the mABC-C and SRS at baseline (n active = 21, n control = 15) and 26 participants completed them at follow-up (n active = 14, n control = 12). 30 participants received the allocated intervention (Fig. 3). On average, participants in the active group had an attendance rate of 82.6% while participants in the control group had an attendance rate of 61.7%. There was only one protocol deviation for a participant in the active group. This participant was lost to follow-up and did not provide post-assessment data. However, they re-engaged the study team and were given the opportunity to participate in the dance classes with the control group. The pre-assessment data for this participant is included in the intention-to-treat analysis and they have been counted as not having received the allocated intervention.

There was no significant change in either the active group (*n* = 21, *p* = 0.11) or the control group (*n* = 15, *p* = 0.82) on the mABC-C total score, although the active group showed a positive trend. The active group displayed a significant improvement on the “movement in an unpredictable environment” domain of the mABC-C (*n* = 21, *p* = 0.05), while the control group did not show any significant change in this domain (*n* = 15, *p* = 0.64). A summary of the data can be seen in Table 3.

**Table 3:** Descriptive Statistics for Study Variables

Baseline characteristic	Pre-intervention		Post-intervention		Change	*p*
	M	SD	M	SD		
mABC-C – unpredictable environment						
Active	19.49	1.79	16.25	2.00	-3.24	0.05*
Control	18.62	2.19	19.45	2.30	0.82	0.64
mABC-C – predictable environment						
Active	11.22	2.15	10.07	2.27	-1.15	0.37
Control	10.97	2.63	10.82	2.69	-0.15	0.91
mABC-C - total						
Active	30.70	3.69	26.38	3.98	-4.32	0.11
Control	29.59	4.51	30.23	4.66	0.64	0.82
SRS – total						
Active	137.54	6.51	134.50	6.88	-3.04	0.44
Control	127.98	7.97	126.66	8.16	-1.32	0.76
SRS – social communication						
Active	45.92	2.34	44.76	2.53	-1.15	0.50
Control	42.05	2.86	41.13	2.96	-0.92	0.62
SRS - social awareness						
Active	13.61	0.83	13.90	0.97	0.29	0.75
Control	11.88	1.02	12.53	1.09	0.65	0.52
SRS - social cognition						
Active	24.21	1.14	24.33	1.23	0.12	0.88
Control	21.19	1.40	22.34	1.44	1.15	0.19
SRS - social motivation						
Active	21.13	1.55	19.46	1.67	-1.67	0.15
Control	20.68	1.89	19.99	1.96	-0.68	0.58
SRS - restricted and repetitive behavior						
Active	32.64	1.59	32.15	1.69	-0.49	0.63
Control	32.26	1.94	30.93	2.00	-1.34	0.23

Table 3*Displays total scores and relevant subscales for the mABC-C and the SRS 2. A reduced score is considered an improvement on these measures*

**Fig. 4:**
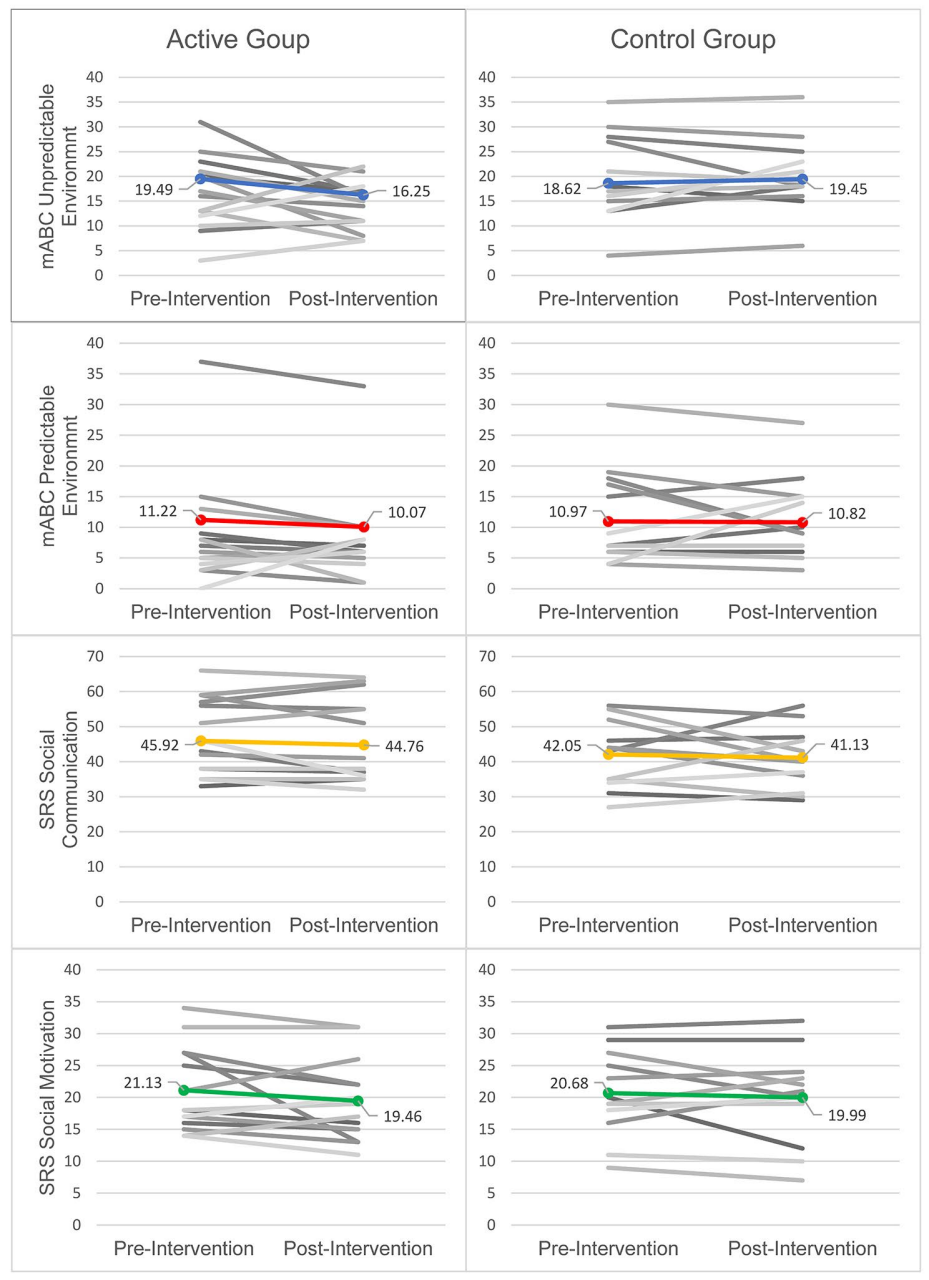
Individual and Mean Scores for mABC-C and SRS Subscales. Figure 4 *Individual scores (grayscale) and means calculated using linear mixed-effects modeling (color) are plotted for subscales of the mABC-C and the SRS. The social communication and social motivation subscales of the SRS are displayed to visualize the change in scores*

Across the domains of the SRS, the active group demonstrated positive trends for improvement in social communication and social motivation, neither of which were significant. We did not observe any notable changes in social awareness, social cognition, or restricted interest and repetitive behavior for the active group. There were no significant changes across any of the domains nor the total score of the SRS for the control group (Fig. 4).

## Discussion

The aim of this pilot study was to investigate the feasibility and impact of a novel movement-based dance intervention program for children and adolescents with NDD. Consistent with our hypothesis, we found that children in the active group showed a statistically significant improvement in movement in dynamic environments, as measured by the mABC-C, and we observed a trend of improvements in social skills on the SRS.

We measured movement skills that are related to movement in dynamic environments, which are targeted through the EMI program activities. Examples of dynamic movement activities include self-care/classroom skills, ball skills, and PE/recreational skills. Examples of specific items covered in this section of the mABC-C include, “moves body in time with music or other people,” “keeps time to a musical beat by clapping hands or tapping feet,” and, “maintains balance when frequent adjustments are required.” This type of movement occurs in a dance class setting in which students are learning how to express themselves using movement through space and time. As such, the expressive movement component of EMI likely accounts for the improvements to movement in dynamic environments. This finding is in line with other movement-based intervention studies which have found similar results on the mABC-2 when assessing the impact of their program on motor skills [20]. Motor skills are integral to many developmental and behavioral domains because they influence how one interacts with their environment. Namely, better motor skills could enhance or improve opportunities to participate in peer interactions by allowing for participation in a broader range of activities, such as sports or active games. Better motor skills may also lead to an increase in active behavior and exercise, which has benefits for social wellbeing, mental health, and cognitive benefits, including a reduction in sedentary behavior [32]. Importantly, improvements in motor skills reported in this study resulted from a relatively low-intensity program that put minimal burden on participating families. One hour of class per week in an online setting offers a more accessible option for families compared to other interventions, as it does not require transportation for participation. Thus, our findings are promising for the viability of OPA programs for children and adolescents with NDDs.

Changes in social skills were characterized by positive trends for social communication and social motivation that were not statistically significant. Participants were given opportunities for social engagement through one-to-one pairings with buddies, which were reinforced by weeks dedicating greater amount of time to buddy interactions. During group time, the instructor also frequently encouraged participants to verbally share their thoughts or experiences, such as their favorite song or what they did during buddy time. These social interactions likely account for the positive trend in social skills noted on the SRS. This result also mirrors similar works that have investigated the impact of OPA on social skills, which have yielded a mix of significant and non-significant results [19]. As will be further discussed in study limitations, several factors such as small sample size and heterogeneity of the participant sample may have impacted our ability to measure significant change in these areas. Considering the impact that negative social interactions can have on individuals with NDD [15], future research is warranted to investigate the impact of OPA programs on social skills and subsequent changes in quality of life. For example, it is possible that positive social interactions during OPA programs increase both one’s motivation to interact with peers and the effectiveness of the communication, which in turn could reduce feelings of loneliness [16].

One strength of the present study was the enrollment of individuals with varying NDDs and co-occurring diagnoses, which served to increase the generalizability of the program. Across participants of EMI, there were diagnoses of autism, ADHD, cerebral palsy, genetic syndromes, and other disabilities. As participants included males and females between the ages of 4 and 17 with no prerequisite for dancing ability, it follows that a wide range of children and adolescents with a diagnosed NDD can benefit from the presented program. This flexibility could be attributed to the training given to buddies, which emphasized adaptable teaching for the specific needs of each student. Furthermore, the decision to use a student-supported structure for class instruction increases the accessibility of the program by allowing for one-to-one pairings between buddies and dancers while remaining cost-free to families who participate.

We acknowledge that there were also several limitations in our implementation, which should be considered when interpreting the results. As a pilot study, our sample size was small and may have limited our ability to identify all effects. Furthermore, the heterogeneity of our participant sample, while positive for the reach and impact of the EMI program, may have constrained our ability to measure significant results related to social communication and social motivation. The reliance on parent reported diagnoses rather than medical records or clinical assessments is another potential limitation. In addition, the quality of our data was affected by a high amount of attrition in the second half of the study, which led to missing data. These factors could lead to a degree of sampling bias in our study population, although it is likely that global changes in the pandemic played a significant role in engagement levels, in part due to a shift back to in-person social, educational, and leisure activities after a prolonged period of these activities being restricted. Several families who did not complete the study reported that they wanted to travel or have their child return to other programs that had been on a hiatus during the pandemic. Indeed, class attendance rate, which was roughly 80% for the active group when COVID-19 restrictions remained in effect, dropped to nearly 60% for the control group after many restrictions were lifted. Finally, it was necessary for our protocol to be transitioned to fully remote due to the pandemic, which did not allow us to conduct direct measures of participants in person. Direct assessment of more detailed motor skills and social skills may have allowed us to detect changes secondary to participation in the dance intervention. Despite opportunities for one-on-one engagement, the program’s effects on social engagement may have also been attenuated due to the online format of the classes.

## Conclusion

In this pilot study, we demonstrate the feasibility of developing and implementing an online dance intervention for individuals with NDDs. Furthermore, this intervention shows benefits in motor skills after a 10-week period with a dose of 1 h per week. Moving forward, we are utilizing direct standardized and quantitative measures of motor skills and social communication to further examine the impact of this dance intervention. Future studies will include an IQ assessment to understand whether this differentially affects the results of the intervention. Future work could also assess the impact of EMI participation on teachers and buddies in order to provide further insight into the efficacy of this approach. Our preliminary results support the growing body of research that OPA is a promising intervention for motor skills among children and adolescents with NDDs.

## Data Availability

The datasets used and/or analyzed during the current study are available from the corresponding author on reasonable request. Full details of the trial protocol version that is reported in this paper can be found on the ClinicalTrials.gov Protocol Registration System, available at https://clinicaltrials.gov/study/NCT04762290?tab=history&a=3.

## Abbreviations

ADHD: Attention deficit/hyperactivity disorder
AIR-P: Autism Intervention Research Network on Physical Health
EMI: Expressive Movement Initiative
HRSA: Health Services and Resources Administration
mABC-C: Movement Assessment Battery for Children Checklist
mABC-2: Movement Assessment Battery for Children – second edition
DCD: Developmental coordination disorder
NDD: Neurodevelopmental disability
OPA: Organized physical activity
SRS: Social Responsiveness Scale
UCLA: University of California, Los Angeles
